# Algorithmic fairness in AI-based fitness advice: evaluating socioeconomic bias in county-contextualized physical activity prescriptions

**DOI:** 10.3389/fpubh.2026.1844470

**Published:** 2026-08-26

**Authors:** Hegui Bao, Tianle Yu, Junyue Wang, Han Yin, Yuan Gao, Yong Mao

**Affiliations:** 1 East China University of Science and Technology, Shanghai, China; 2 Shanghai Normal University, Shanghai, China; 3 University of Birmingham, Birmingham, United Kingdom; 4 Shanghai University, Shanghai, China; 5 Department of Acupuncture and Massage, Zhejiang Rehabilitation Medical Center, Hangzhou, Zhejiang, China; 6 Jimei University, Xiamen, Fujian, China

**Keywords:** algorithmic fairness, large language models, physical activity recommendation, public health, socioeconomic disparity

## Abstract

**Background:**

Large language models (LLMs) can significantly broaden access to physical-activity guidance. However, advice that implicitly assumes available financial resources, reliable transportation, specialized equipment, or local facilities can be difficult for individuals in resource-constrained environments to act upon. We evaluated whether such resource assumptions systematically vary across socioeconomic settings when underlying health needs remain fixed.

**Methods:**

We developed a county-aware, matched-counterfactual auditing framework integrating U.S. public health and socioeconomic data, synthetic patient profiles, and structured LLM outputs. To evaluate resource burden, we constructed a transparent access-cost proxy that renders each coded component directly inspectable. We evaluated accessibility-aware prompting, output reranking, alternative weighting schemes, and a bounded three-model comparative panel (including DeepSeek) to audit and mitigate socioeconomically driven bias.

**Results:**

In the full DeepSeek model panel, accessibility-aware prompting reduced the access-cost proxy gap between high- and low-socioeconomic status (SES) counties from 0.306 to 0.139. Subsequent output reranking maintained this narrowed gap while simultaneously improving coarse alignment with physical-activity volume and intensity guidelines. Sensitivity analyses using alternative weighting schemes preserved the overall comparative ordering, while the three-model comparison demonstrated model-specific variations in resource-assumption responses.

**Discussion:**

This study establishes a reproducible framework that connects equity-oriented health-recommender principles to traceable output auditing and targeted bias mitigation. By offering a transparent approach to evaluating implicit resource assumptions, this work provides researchers and practitioners with an actionable foundation for downstream expert, user, and implementation validation.

## 1 Introduction

Large language models (LLMs) are increasingly used as the first interface for everyday health-adjacent decisions. In consumer settings, they are already asked to generate walking plans, home exercise routines, weight-management suggestions, and general wellness advice, often with an immediacy that makes them feel more accessible than traditional professional channels. That accessibility is part of their promise. Physical-activity guidance is unevenly available across communities, and many users do not have timely access to coaches, trainers, or clinicians for low-acuity questions about exercise planning. An LLM can therefore appear to reduce informational barriers. Yet the same system can also carry unspoken assumptions about disposable income, neighborhood infrastructure, work schedules, transportation, or access to recreational facilities. In a domain such as physical activity, those assumptions are not secondary. They shape whether a recommendation is realistically actionable.

This paper examines accessibility-related differences in AI-generated fitness advice within the broader framework of algorithmic fairness. Conceptually, accessibility concerns whether a recommendation can be acted upon given its cost, transport, equipment, facility, and time demands. Empirically, we operationalize one part of that construct as a transparent output-level proxy of explicit resource requirements. Burden denotes the combined practical demands encoded by those features. Equity is the normative objective of avoiding systematic, remediable differences in access to beneficial opportunities, whereas algorithmic fairness is the broader assessment of whether an algorithm distributes benefits, harms, opportunities, and burdens appropriately across people and groups. The present audit measures socioeconomic differences in the proposed proxy; this reproducible operationalization informs one dimension of fairness without replacing patient-reported or behaviorally validated accessibility. The central empirical question is whether recommendations change systematically when social and geographic context changes while underlying health need remains fixed. This problem sits at the intersection of several active lines of research. Work on recommender-system fairness has established that relevance or predictive utility is an incomplete evaluation target when algorithmic outputs distribute opportunity, burden, or exposure unevenly across people and groups (1, 2). In parallel, recent frameworks for LLM auditing argue that meaningful fairness evaluation requires matched prompts, controlled counterfactual variation, and harm metrics tailored to the actual use case rather than generic bias screens (3, 4). Public-health evidence points in the same direction. U.S. adult activity guidelines are formally uniform, but the feasibility of meeting them is not: physical inactivity, transportation constraints, environmental resources, and healthcare access remain strongly socially patterned (5, 6). Taken together, these literatures suggest that plausible-seeming advice may still distribute practical burden unevenly across users. Clinical appropriateness must be assessed separately. Recent work in health-related LLM evaluation makes this concern harder to dismiss. Studies in medical decision support and biomedical informatics show that language models can reproduce or amplify sociodemographic patterns in diagnostic reasoning, triage, and recommendation behavior (7–9). What remains underexplored is the lower-acuity but higher-frequency domain of physical-activity recommendation. That omission matters. “Fitness advice” is often treated as benign when compared with diagnosis or medication guidance, but it can still encode assumptions about cost, schedule flexibility, disability accommodation, environmental safety, and access to exercise-supporting infrastructure. Because this is exactly the kind of advice that consumer-facing AI systems are likely to deliver at scale, it is an appropriate and policy-relevant target for audit.

To study the issue, we develop a county-aware audit that combines CDC PLACES indicators, American Community Survey variables, matched synthetic user profiles, a live DeepSeek endpoint, and structured scoring of recommendation burden and accessibility-related resource assumptions. The study provides a controlled algorithm audit that complements clinical validation and addresses an operationally important question: when health need is held constant, does the model encode comparable resource requirements across different socioeconomic contexts?

Our contribution is threefold. First, we introduce a public-data-driven, matched-counterfactual setting for auditing socioeconomic differences in the resource assumptions of AI-generated fitness advice while holding represented health need constant. Second, we replace impressionistic judgments with a transparent, decomposable proxy built from observable cost, transport, equipment, and facility features, making every score traceable to specific recommendation content. Third, using live model outputs, we show that accessibility-aware prompting reduces the proposed access-cost score and narrows its county-level socioeconomic gap in the full DeepSeek panel, whereas guideline-constrained prompting by itself does not remove the pattern. Together, these properties establish the framework as a reproducible first-stage screen and identify specific outputs for expert or user-centered follow-up, which can test how closely proxy differences correspond to lived accessibility.

## 2 Related work

The present study sits at the intersection of fairness-aware recommendation, application-specific LLM auditing, and public-health inequality research. In recommender systems, fairness has long been framed not simply as equal predictive quality but as the equitable distribution of exposure, burden, and opportunity across users and groups (1, 2, 10). That broader view is especially relevant when the recommendation itself imposes material demands. A plan that assumes paid facilities, reliable transport, or abundant leisure time may be “relevant” in a narrow semantic sense while still being structurally exclusionary. Work on collaborative filtering and ranking has made this point in several complementary ways, including explicit fairness objectives for recommendation, pairwise ranking disparities, and exposure-sensitive evaluation (11, 12). Our setting differs from classical ranking, but the underlying concern is the same: recommendation quality cannot be separated from who is realistically able to act on the recommendation.

Recent fairness research for LLMs has moved toward task-specific audits that perturb socially salient attributes under controlled prompt templates and then evaluate harms in terms meaningful for the application at hand (3, 4, 13). This move matters because generic toxicity or stereotype tests are poorly matched to health-adjacent advice systems, where the most consequential harm may be a quiet redistribution of feasibility rather than overtly offensive language. Tooling ecosystems such as AIF360 and Fairlearn are also relevant here, not because they solve the generative setting directly, but because they helped normalize the idea that fairness claims should be tied to explicit metrics, diagnostic slices, and mitigation comparisons rather than anecdotal examples alone (14, 15). We adopt that general philosophy while tailoring the audit to structured physical-activity recommendations.

The mitigation literature provides an additional point of contrast. Prompt-only interventions are attractive because they are easy to implement, but they often remain shallow when compared with more deliberate self-correction or output-selection mechanisms. Work on Constitutional AI, Self-Refine, Reflexion, and LLM-based evaluators illustrates a broader pattern: lightweight post-generation constraints or reflective selection can alter model behavior in ways that are not reducible to a single static instruction (16–19). Our reranking baseline is intentionally simpler than those methods, yet it belongs to the same family of ideas. Instead of asking a model to “be fair” and trusting the answer, we compare candidate outputs under explicit accessibility and guideline criteria.

The public-health motivation is equally important. Socioeconomic conditions shape physical-activity opportunities well-before an AI system enters the loop. County- and population-level data show persistent gradients in leisure-time inactivity, obesity, insurance instability, transport constraint, and related correlates of exercise feasibility (6, 20, 21). Environmental-justice research further shows that green space, blue space, and other health-supporting resources are unevenly distributed across social groups (22, 23). In parallel, health-oriented LLM evaluations have shown that demographic and socioeconomic signals can affect diagnostic framing, treatment recommendation, and health-related advice (7–9, 24). Emerging work on fairness in LLM-based recommendation systems points in the same direction (25, 26). What remains underexplored is a focused audit of consumer-facing exercise advice, where the practical question is not merely whether the recommendation is safe, but whether it embeds unequal assumptions about cost, access, and day-to-day implementability. That is the gap this paper addresses.

Two complementary perspectives sharpen the theoretical positioning of this audit. The socioecological perspective for equity-oriented health recommender systems treats health behavior as the product of interacting individual, interpersonal, community, and societal influences and calls for structural barriers and localized context to enter personalization (27). Our county-contextualized matched design makes the representation of the individual–community interface directly auditable in model outputs: represented health need remains fixed while the county context in which advice must be enacted varies, and shifts in explicit resource demands are traced at the output level. The HiCARE framework complements this perspective through human-centric hybrid integration with clinical expertise, broad stakeholder inclusion, and a learning-driven approach centered on continuous skill development (28). By pairing decomposable automated screening with a separate guideline-alignment check and clear expert- and user-validation pathways, the present framework converts these equity and responsible-AI principles into a reproducible audit-and-mitigation design for generative fitness advice. This theoretical link motivates the matched-counterfactual strategy summarized next.

## 3 Methodology overview

The study uses a controlled matched-counterfactual algorithm audit. This design is appropriate for the research questions because represented health need is held constant while socioeconomic and place-based context varies, allowing context-associated changes in observable resource assumptions to be isolated within the audited prompts. Public county data provide ecologically grounded contextual anchors without exposing patient records, while a common structured output schema and consistently applied intervention conditions support reproducible comparison.

The workflow proceeds chronologically. First, CDC PLACES and ACS indicators are merged to construct county contexts and SES strata. Second, those contexts are paired with synthetic health archetypes to form matched counterfactual profiles. Third, models generate structured physical-activity advice under raw, guideline-constrained, and accessibility-aware prompts. Fourth, the pipeline parses the structured fields and computes burden, safety, personalization, and coarse guideline-alignment measures. Fifth, the output-layer reranker uses the scored guideline-constrained and accessibility-aware candidates to supply the fourth evaluated condition, after which group and counterfactual proxy gaps are estimated. Sixth, score-sensitivity and bounded cross-model analyses test comparative stability and model heterogeneity. The sections that follow mirror this logic: Study Design defines the questions, data, profiles, and outputs; Experimental Setup specifies the interventions, measures, comparisons, and implementation; Results reports the primary condition comparison and then resolves RQ1–RQ3 through group, counterfactual, cross-model, and sensitivity analyses.

## 4 Study design

### 4.1 Research questions

The study is organized around three linked research questions.

**RQ1**. When represented health need is held constant, do LLM-generated physical-activity prescriptions vary across matched users with different county socioeconomic contexts?

**RQ2**. Which aspects of socioeconomic context—particularly income, insurance stability, and county environment—show the largest differences in the proposed access-cost proxy?

**RQ3**. Can accessibility-aware prompting and output-layer reranking reduce gaps in the proposed access-cost proxy while retaining structured and individualized recommendations?

**Figure 1:**
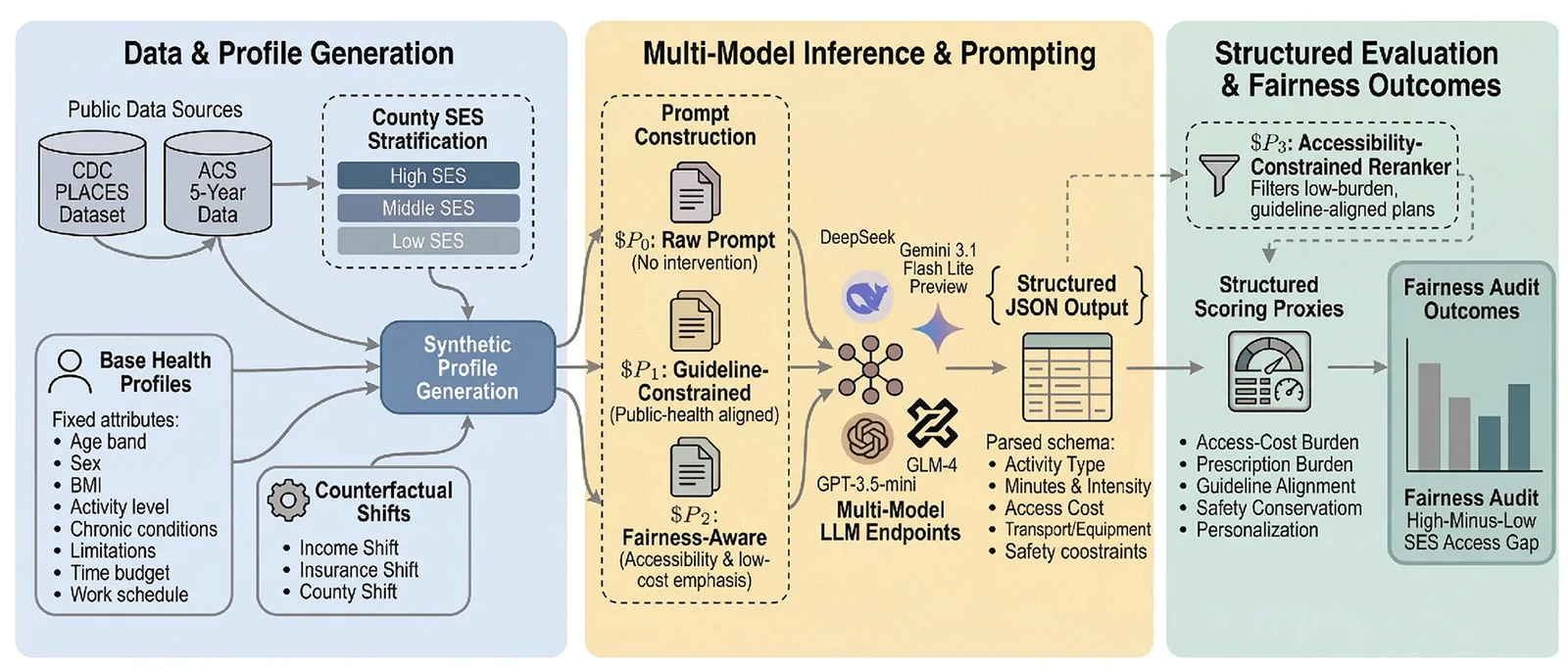
Core architecture of the county-aware audit framework.

Because the counterfactual comparison holds the represented health attributes constant, a systematic shift in the access-cost proxy associated with socioeconomic context is treated as a potential proxy-defined accessibility disparity. This design does not by itself classify every between-profile difference as discrimination or establish clinical equivalence. The overall county-aware audit pipeline that links county data, matched profiles, structured generations, and downstream scoring is summarized in Figure 1.

### 4.2 Public data backbone

The empirical backbone combines CDC PLACES county estimates with 2023 ACS 5-year county indicators. PLACES provides adjusted prevalence estimates for no leisure-time physical activity, obesity, poor mental health, insurance access, food insecurity, housing insecurity, and lack of transportation. ACS contributes median household income, poverty, unemployment, and educational attainment. We use these sources because they jointly capture both health-relevant need and the surrounding socioeconomic context in which physical activity advice would be acted upon.

**Figure 2:**
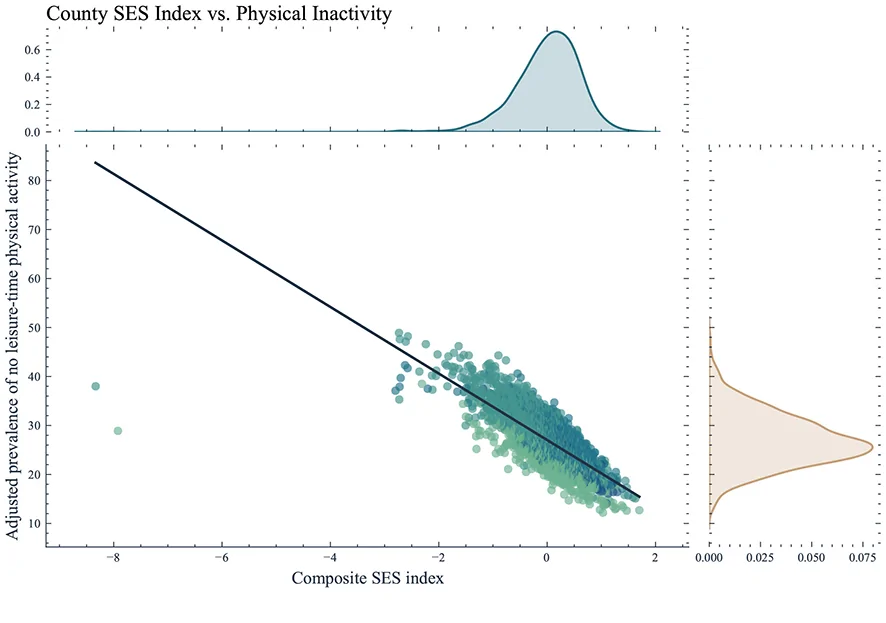
County-level relationship between the composite SES index and adjusted prevalence of no leisure-time physical activity. Each point denotes one county in the cleaned analysis panel (*n* = 2, 956). Lower-SES counties are systematically associated with higher inactivity prevalence. Public data sources: CDC PLACES county estimates and ACS 2023 county-level socioeconomic indicators.

**Figure 3:**
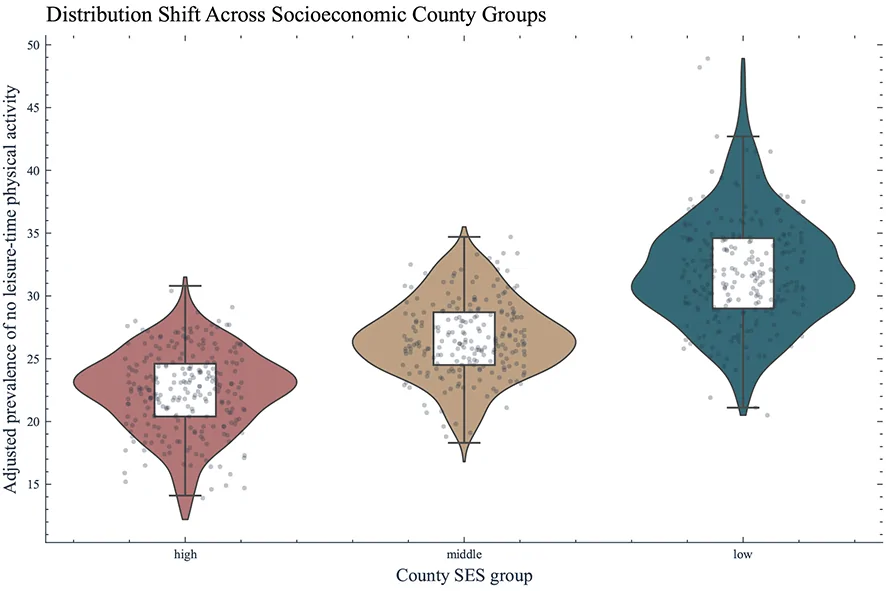
Distribution of county-level inactivity prevalence across SES terciles. The low-SES tercile is shifted upward and remains more dispersed, reinforcing the need for an accessibility-sensitive audit of generated recommendations.

The county-level SES index is defined as the average of standardized income and education minus standardized poverty, unemployment, and uninsurance. Counties are then assigned to low-, middle-, and high-SES terciles. This formulation is intentionally transparent. The goal is not to claim a definitive deprivation index, but to create an interpretable socioeconomic gradient that can anchor a fairness audit and remain legible in the manuscript. The merged county panel contains 2,956 counties after cleaning and one-to-one geographic joins. Table 1 summarizes the county backbone, synthetic profile design, and total audit workload. In addition to the full panel, the study also uses a balanced 36-profile subset for the completed cross-model comparison reported later in the paper. The county gradient motivating this design is also visible in the merged panel itself: Figure 2 shows a clear inverse relationship between county SES and inactivity prevalence, while Figure 3 shows that the low-SES tercile is both upward-shifted and more dispersed.

**Table 1:** Data backbone, synthetic profile design, and audit workload.

Component	Value
County units in merged panel	2,956
Low-SES counties	985
Middle-SES counties	985
High-SES counties	986
Full synthetic profiles	108
Cross-model panel profiles	36
Health archetypes	4
Counterfactual scenario types	3
Full-audit conditions scored	4
Full-audit recommendation rows	432
Cross-model families completed	3
Cross-model recommendation rows	324

### 4.3 Synthetic profile construction

The audit uses matched synthetic profiles rather than real patient records. Each profile specifies age band, sex, baseline activity level, body mass index band, chronic-condition marker, injury limitation, weekly time budget, and work schedule constraint. These health and lifestyle attributes define the underlying exercise-planning problem. County context, income band, education band, insurance stability, and activity-environment access are then attached to the base profile so that social context can vary independently of health need. Four health archetypes are used to cover common exercise-planning situations: a sedentary overweight adult, an office worker with hypertension risk, a middle-aged adult with knee limitations, and an older beginner with low baseline activity.

The design follows a deliberately counterfactual logic. For a matched set of prompts, the represented health profile remains constant while one dimension of social context changes, most often income band, insurance status, or county context. A change that responds to an explicit health limitation or stated preference can reflect appropriate personalization. By contrast, a systematic increase in cost, transport, equipment, or facility burden associated only with socioeconomic context is treated here as a potential proxy-defined accessibility disparity. This operational rule identifies patterns for audit; it does not independently establish discriminatory intent, clinical harm, or complete algorithmic unfairness. To avoid cherry-picking extreme places, county anchors are selected near the median physical inactivity rate within each SES tercile rather than at the tails of the distribution. The final full-scale prompt set covers 108 synthetic profiles and 324 direct model generations across the three primary prompt conditions. The fourth condition reported later in the paper is an output-layer reranking baseline constructed from those candidate recommendations, which brings the full scored audit workload to 432 recommendation records.

### 4.4 Structured recommendation output

The model is required to return valid JSON with fields describing activity type, weekly minutes, intensity, cost level, transport requirement, equipment needs, gym dependence, safety cautions, and personalization notes. That structured schema supports reproducible parsing and makes the downstream analysis less dependent on impressionistic reading of free-form text. The original recommendation text is still retained for qualitative inspection and for the illustrative appendix case, but the primary analysis relies on structured outputs that can be scored consistently across prompts and mitigation conditions.

Together, these design elements specify what is held constant, varied, and observed. The next section defines how recommendations are generated, scored, compared, and stress-tested.

## 5 Experimental setup

### 5.1 Prompt conditions and mitigation

The core audit compares three prompt conditions that differ in how strongly they constrain the model. The raw condition, denoted P0, asks for standard fitness advice without an explicit accessibility intervention. The guideline condition, P1, adds instructions to align the recommendation with public-health-style physical-activity guidance. The accessibility-aware condition, P2, builds on the guideline prompt by explicitly discouraging socioeconomic stereotyping and requesting low-cost, low-resource alternatives whenever possible. The full empirical results are first established on the complete DeepSeek panel so that every matched profile is observed under the same model family and output schema.

Prompt-only mitigation is not the only intervention we evaluate. To move beyond the weakest version of prompt engineering, we introduce a lightweight post-generation baseline, P3, that reranks candidate recommendations drawn from the guideline-constrained and accessibility-aware conditions. The selector prefers outputs that satisfy a minimal guideline-alignment rule and then chooses the lower access-cost proxy candidate, breaking ties in favor of higher personalization. Because P3 selects only between the P1 and P2 outputs generated for the same profile, it is evaluated as a fixed two-candidate reranking baseline. The audit does not test whether the same selection behavior would hold for larger, independently sampled, or differently constructed candidate pools. This procedure is deliberately simple: its role is not to claim a new alignment algorithm, but to test whether explicit output-layer selection changes the access-cost-proxy–alignment trade-off relative to prompt wording alone.

### 5.2 Structured scoring and guideline checks

After parsing the JSON output, the pipeline computes four structured audit proxies. The *prescription burden score* aggregates time commitment, intensity, equipment count, and dependence on gyms or outdoor infrastructure. The *access-cost burden score* is an investigator-defined proxy that summarizes coded cost level, transport requirement, gym dependence, and equipment count. These components are directly observable in the structured recommendation, separately inspectable, and actionable for prompt or output-layer mitigation. The *safety conservatism score* summarizes the number of cautions and whether the model escalates to professional referral. The *personalization score* reflects how clearly the recommendation adapts to the user's stated constraints.

The four access-cost domains were selected for documented relevance to modifiable barriers and direct observability in structured recommendations. A systematic review of reviews identifies affordability, public transport, equipment availability, and access to activity settings among practical barriers and facilitators examined in physical-activity research (29); environmental-equity research further documents socially patterned access to exercise-supporting spaces (22, 23). Cost and transport capture financial and mobility demands; equipment and gym dependence capture material and facility reliance. This literature-grounded architecture makes every component auditable and actionable. The separate prescription burden score captures time, while neighborhood safety, disability accommodation, social support, and local availability offer clear extension points for user- and place-level assessment.

These quantities are structured audit proxies rather than clinically validated outcome measures. Their purpose is to make differences in recommendation burden inspectable and reproducible across hundreds of matched prompts. The access-cost weights give greatest emphasis to direct monetary and transport demands, followed by gym dependence and equipment count, because these features represent distinct, observable resource assumptions in the recommendations. The metric's transparency is a deliberate strength: unlike a latent or learned score, it can be decomposed into its contributing features, independently recalculated, and directly connected to mitigation choices. The weights remain heuristic rather than a validated scale of lived burden, so we test six alternative specifications that vary the contributions of transport, equipment, and gym dependence. This combination of feature-level traceability and score-sensitivity analysis supports comparative audit use, while external expert and user assessment remains necessary to establish how well the proxy represents experienced accessibility. We also compute a binary volume-and-intensity alignment check based on thresholds in the U.S. Physical Activity Guidelines for adults. This coarse check can identify plans that miss basic activity-volume or intensity thresholds, but it does not evaluate frequency distribution, progression, individualization, contraindications, or overall clinical appropriateness.

### 5.3 Access-cost proxy-gap analysis and sensitivity checks

The main analysis compares average burden, personalization, and the volume-and-intensity alignment check across county SES groups within each condition. The central disparity statistic is the high-minus-low SES gap in the proposed access-cost proxy because it provides a concise, reproducible summary of how coded resource assumptions vary across social contexts. The analysis is then extended with counterfactual summaries organized by income shift, insurance shift, and county shift, allowing us to ask which aspects of the social context drive the strongest proxy differences. Because the burden metrics are heuristic by construction, the paper also includes a systematic score-sensitivity analysis. We re-estimate the access-cost proxy gap under six alternative weighting schemes that increase or decrease the contribution of equipment and transport, including a binary-access variant that places more emphasis on gym dependence than on itemized equipment counts. This analysis tests whether the comparative ordering of the prompt conditions is stable under plausible scoring choices. Stability across these alternatives supports the robustness of the between-condition pattern, while the absolute burden values retain their proxy interpretation.

### 5.4 Implementation pipeline and cross-model extension

The implementation is fully tied to the accompanying repository. The same workflow downloads and preprocesses public county data, generates matched synthetic profiles, serializes prompts, executes resumable API calls, scores the resulting recommendations, exports the tables and figures used in the manuscript, and packages the manuscript for local or Overleaf compilation. That end-to-end structure matters for interpretation because the paper is not built from hand-curated screenshots or a small set of cherry-picked examples.

To examine whether the single-endpoint pattern appears beyond the full DeepSeek panel, the study includes a balanced 36-profile cross-model comparison executed through OpenRouter. That panel uses three additional model families, namely openai/ gpt-5.4-mini, google/ gemini-3.1-flash-lite-preview, and z-ai/ glm-5. This bounded comparison applies a common prompt schema and the same structured scoring layer. Its purpose is to document directional agreement and heterogeneity within these 36 profiles, not to estimate provider-wide or universal generalizability.

The Results section follows this workflow: it first reports intervention effects, then examines socioeconomic gaps and their drivers, before testing model heterogeneity and score-weight sensitivity.

## 6 Results

### 6.1 Prompting and reranking change encoded resource requirements in different ways

Table 2 summarizes the main audit outcomes for the full DeepSeek panel. The four conditions do not merely produce stylistic variants of the same recommendation. They shift the encoded resource requirements of the output in substantively different ways. Under the guideline-constrained prompt, mean prescription burden rises from 4.922 to 5.316, which suggests that the instruction to follow public-health guidance is interpreted primarily as a signal to increase recommended activity load. Under the P2 accessibility-aware prompt, by contrast, the mean access-cost proxy falls from 2.139 to 1.824 while mean personalization remains above 5.1. Relative to the raw prompt, this corresponds to a 14.7% reduction in the average access-cost proxy.

**Table 2:** Main full-scale audit results for the DeepSeek panel.

Prompt	Presc.	Access	Safety	Pers.	**Align**.	Gap
Raw prompt	4.922	2.139	3.755	5.167	0.019	0.306
Guideline	5.316	2.241	3.796	5.167	0.250	0.291
P2 access.-aware	5.005	1.824	3.741	5.111	0.046	0.139
Rerank	5.211	1.861	3.792	5.194	0.250	0.139

Lower access-cost proxy scores indicate fewer coded cost, transport, equipment, and facility assumptions, and a lower high-minus-low proxy gap indicates less variation in those features across county SES contexts.

The reranking baseline reveals an additional trade-off that is not visible from prompt-only comparisons. The P2 accessibility-aware condition produces the lowest average access-cost proxy score, but its volume-and-intensity alignment rate remains low at 0.046. Accessibility-constrained reranking preserves the reduced high-minus-low SES proxy gap of 0.139 while increasing this coarse alignment rate to 0.250. The reranker therefore selects a lower-scoring candidate among outputs that satisfy the minimum volume-and-intensity rule. This demonstrates the proxy's practical value as an explicit output-selection criterion, while the separate alignment criterion preserves the distinction between resource screening and recommendation quality.

### 6.2 County SES gaps in the access-cost proxy remain visible without accessibility constraints

**Figure 4:**
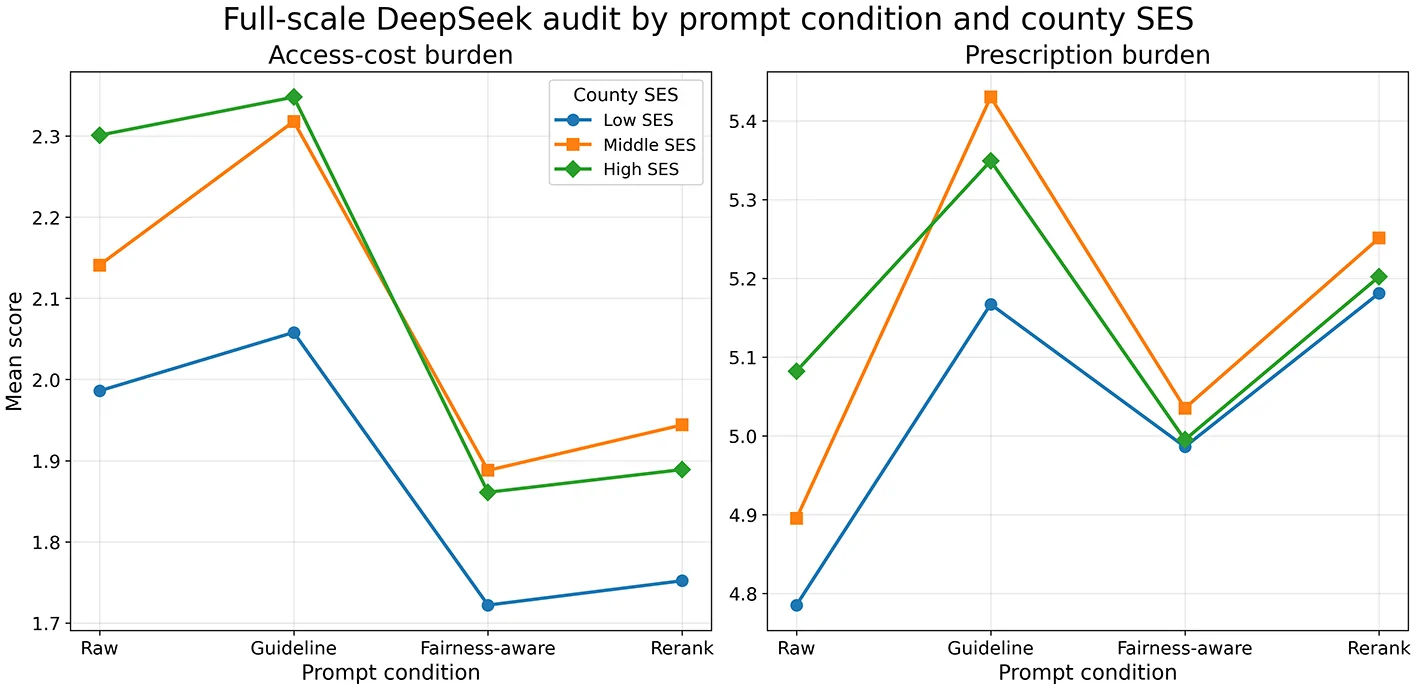
Mean access-cost and prescription-burden proxy scores across prompt conditions, stratified by county SES group. P2 accessibility-aware prompting reduces the proposed access-cost score across all SES terciles, and accessibility-constrained reranking preserves the smaller proxy gap while avoiding the highest average prescription-burden score.

Figure 4 shows the main proxy pattern by county SES group. Under the raw prompt, the mean access-cost proxy is highest for high-SES county contexts and lowest for low-SES county contexts, yielding a high-minus-low proxy gap of 0.306. This finding does not imply that high-SES users are disadvantaged in the broader public-health sense. The gradient shows that the model assigns more resource-intensive recommendations to socially advantaged contexts and lower-cost plans to less advantaged contexts. The audit concern is the context-dependent allocation of coded resource assumptions when represented health need is held constant. Guideline-constrained prompting does not remove that pattern; the corresponding proxy gap remains 0.291. The P2 accessibility-aware condition narrows the proxy gap to 0.139, and reranking retains that gap while shifting more outputs into the volume-and-intensity-aligned subset.

This narrowing is not a trivial artifact of forcing the model toward the same minimal answer in all cases. Under the P2 accessibility-aware condition, mean prescription burden remains at 5.005, and under reranking it remains 5.211, both well-within the range of substantive weekly plans rather than empty boilerplate. Personalization scores also remain high across all mitigation conditions. The model is therefore not becoming uniformly vague or non-committal. Instead, coded resource assumptions are being reallocated: the recommendations remain structured and individualized, but the coded cost, transport, equipment, and facility assumptions become less socially stratified.

### 6.3 Insurance and county context show the largest proxy gaps

**Figure 5:**
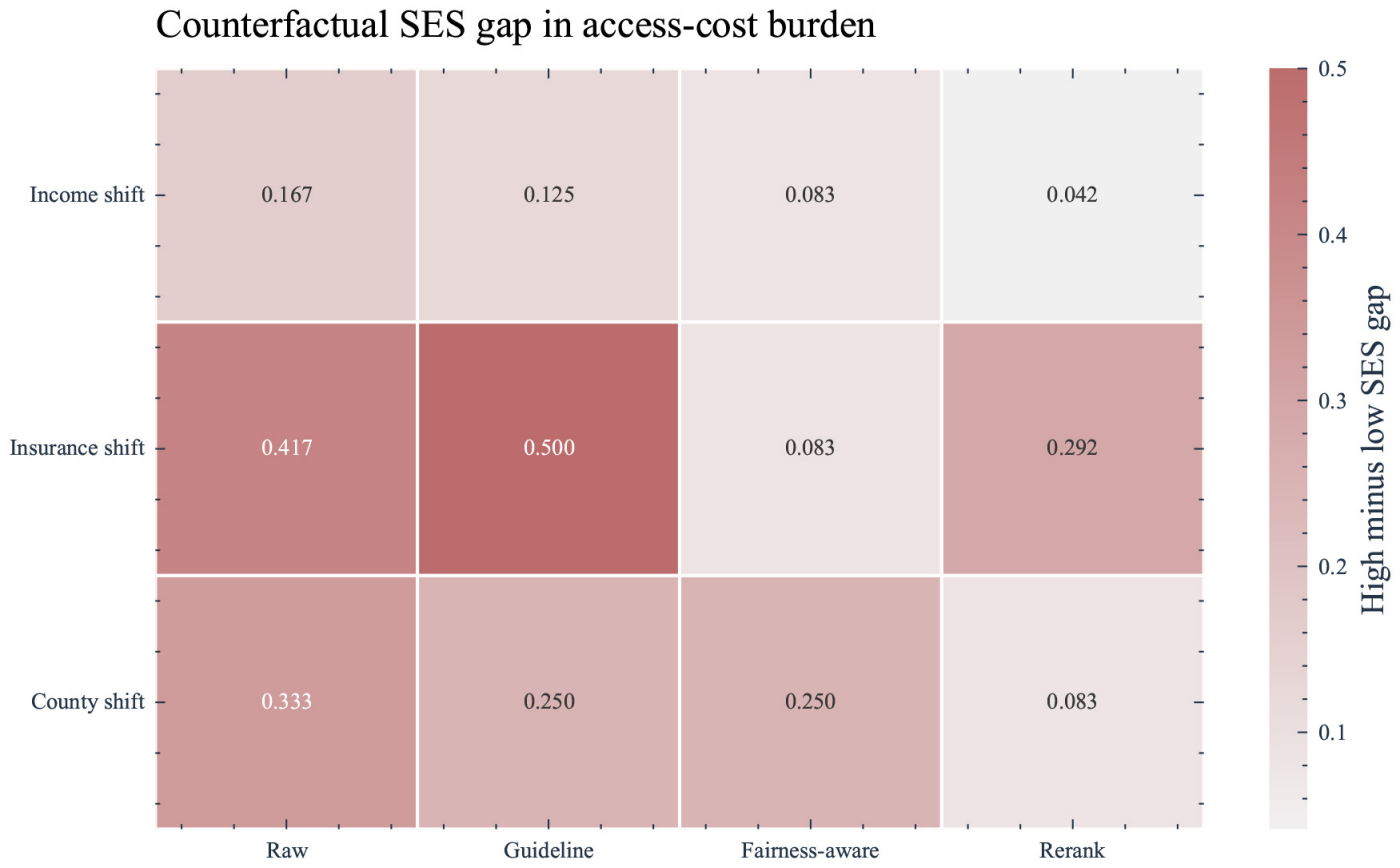
High-minus-low SES access-cost proxy gap across counterfactual scenario types and prompt conditions. Insurance-related shifts exhibit the largest proxy gaps under raw and guideline-constrained prompting, whereas P2 accessibility-aware prompting and reranking compress the gap across all three scenario families.

The next question is whether the observed proxy gap is driven primarily by individual socioeconomic cues or by broader place-based context. Figure 5 reports the high-minus-low SES access-cost proxy gap for each scenario type and prompt condition. Insurance-shift scenarios exhibit the largest proxy gaps under the raw and guideline-constrained prompts, while P2 accessibility-aware prompting and reranking substantially compress them. County-shift scenarios retain residual proxy gaps, which is consistent with the fact that county context bundles several environmental assumptions at once, including activity infrastructure, transport burden, and the plausibility of outdoor or facility-based exercise.

The pattern is not only visible in the aggregate score but consistent across scenario types. For income-shift scenarios, the high-minus-low access-cost proxy gap drops from 0.167 under the raw prompt to 0.083 under the P2 accessibility-aware prompt. For insurance-shift scenarios, the proxy gap falls from 0.417 to 0.083. These shifts support the claim that mitigation changes coded resource assumptions rather than only the tone or surface form of the answer. Their magnitude is interpreted on the proposed proxy scale, not as an equivalent change in users' experienced accessibility.

### 6.4 A bounded cross-model comparison shows heterogeneous responses

**Figure 6:**
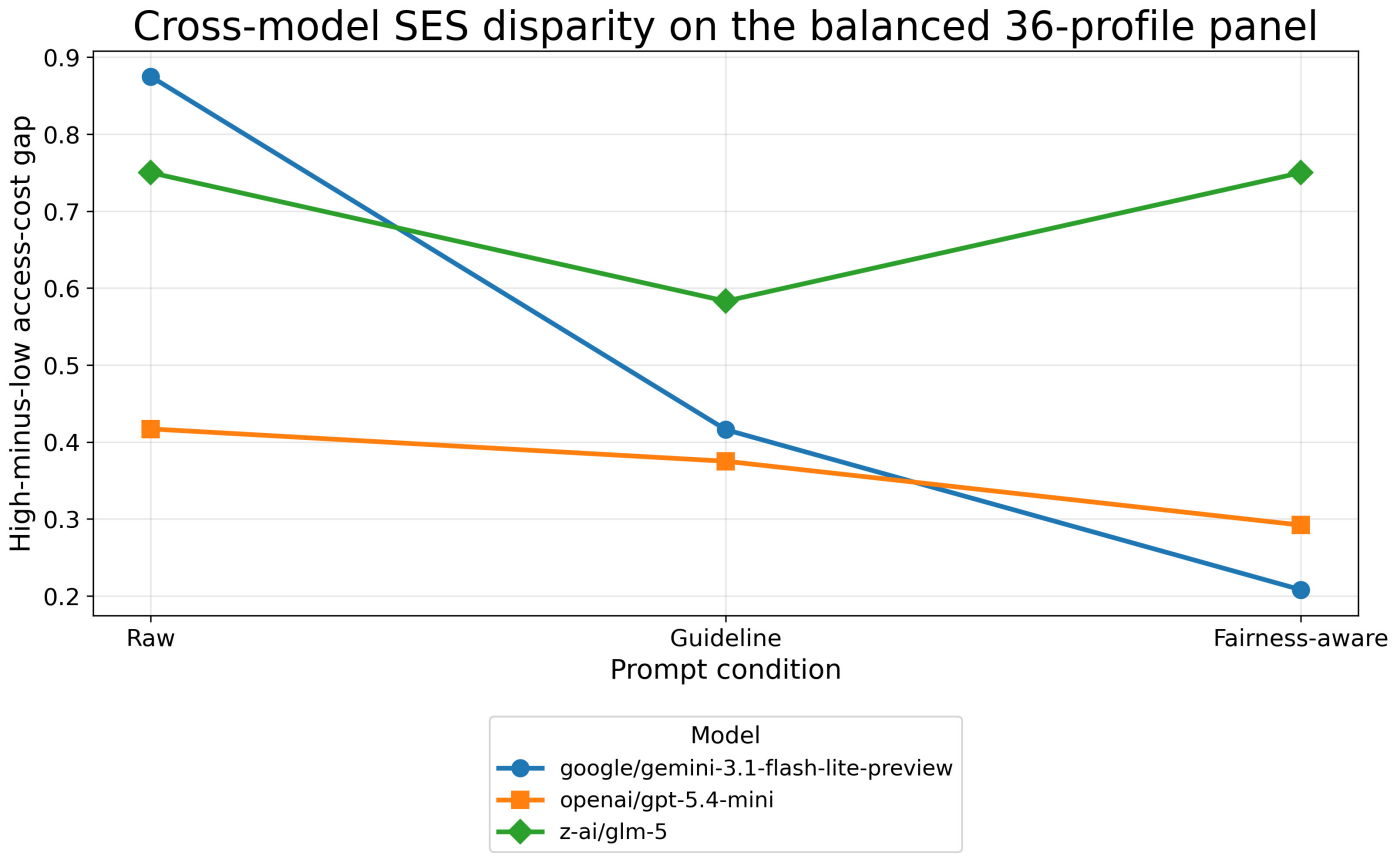
Cross-model comparison of the high-minus-low SES access-cost proxy gap on the balanced 36-profile panel. The P2 accessibility-aware condition narrows the proxy gap for Gemini and GPT-5.4-mini but not for GLM-5, documenting heterogeneous responses within the tested profiles.

The balanced 36-profile comparison examines whether the single-endpoint pattern is also visible under three additional model families within a common evaluation setup. As shown in Table 3 and Figure 6, the raw high-minus-low SES access-cost proxy gap is non-trivial for all three models, and accessibility-aware prompting reduces that proxy gap for two of them. For google/gemini-3.1-flash-lite-preview, the proxy gap falls from 0.875 under the raw prompt to 0.208 under the accessibility-aware prompt. For openai/gpt-5.4-mini, the corresponding reduction is from 0.417 to 0.292. These observations provide a limited directional comparison across the tested profiles; the panel size does not support prevalence estimates or broad claims about model providers.

**Table 3:** Cross-model audit summary on the balanced 36-profile panel.

Model/condition	Access proxy	Presc. burden	Align.	High-low proxy gap
google/gemini-3.1-flash-lite-preview / P0 raw	1.319	5.137	0.250	0.875
google/gemini-3.1-flash-lite-preview / P1 guideline	1.319	5.380	0.250	0.416
google/gemini-3.1-flash-lite-preview / P2 access.-aware	1.181	5.181	0.222	0.208
openai/gpt-5.4-mini / P0 raw	2.694	5.347	0.194	0.417
openai/gpt-5.4-mini / P1 guideline	2.458	5.433	0.250	0.375
openai/gpt-5.4-mini / P2 access.-aware	2.222	5.394	0.167	0.292
z-ai/glm-5 / P0 raw	1.861	4.634	0.194	0.750
z-ai/glm-5 / P1 guideline	2.042	4.917	0.194	0.583
z-ai/glm-5 / P2 access.-aware	1.542	4.479	0.083	0.750

Lower values indicate lower mean proxy scores or smaller high-minus-low proxy gaps.

The heterogeneous result for z-ai/glm-5 further defines the boundary of this comparison. Its mean access-cost proxy is lower under accessibility-aware prompting, while the high-minus-low SES proxy gap remains 0.750. The mean proxy score and between-group proxy gap therefore capture different properties of the recommendations. Across this bounded panel, the prompt intervention changes the proxy gap for some models but not all, supporting model-specific evaluation rather than a universal claim.

### 6.5 The comparative proxy ordering is robust to alternative weighting schemes

**Figure 7:**
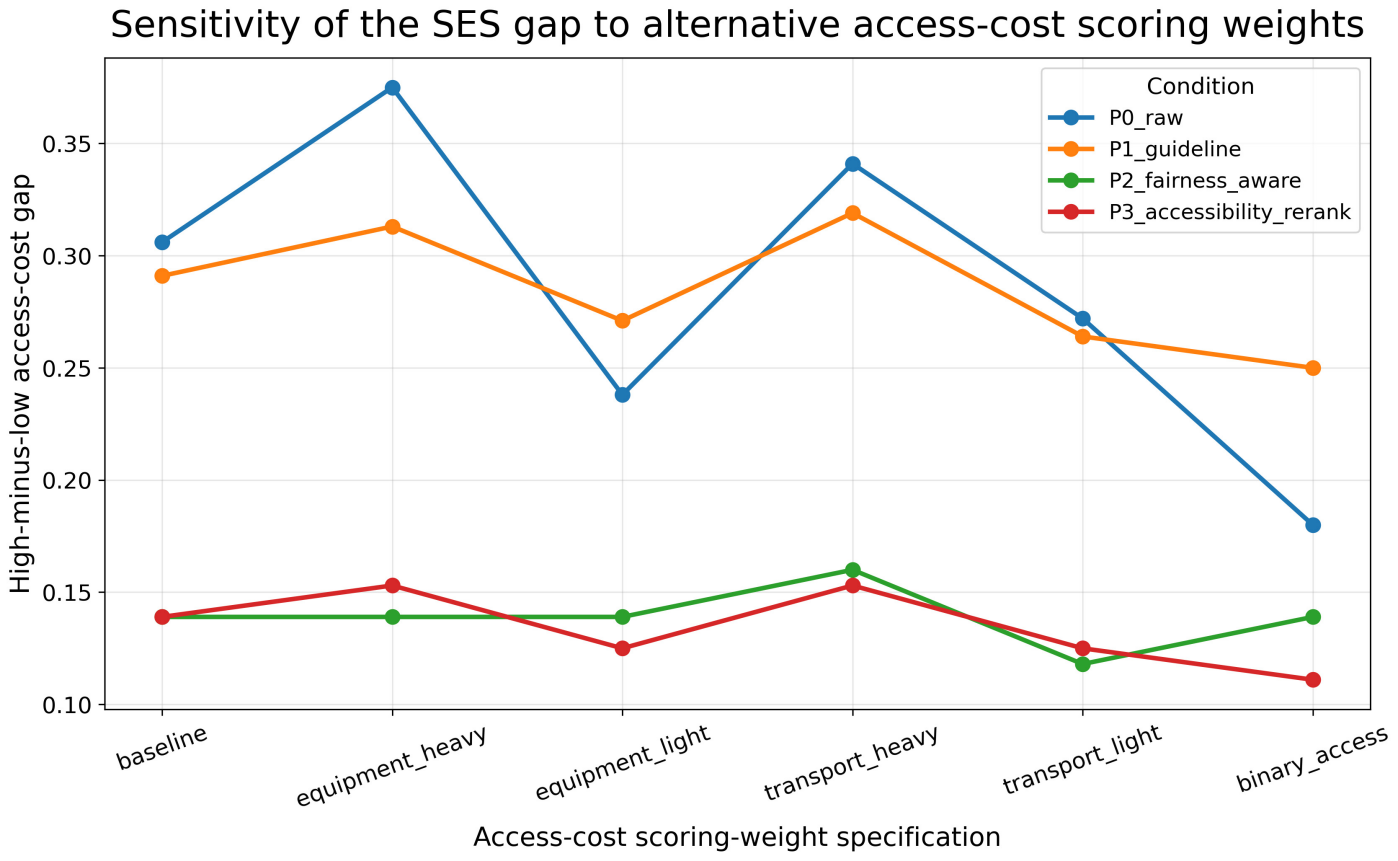
Sensitivity of the high-minus-low SES access-cost proxy gap to alternative scoring weights. The P2 accessibility-aware and reranked conditions remain systematically lower than the raw and guideline-constrained conditions across all tested variants.

The score-sensitivity analysis asks whether the comparative ordering depends on the particular access-cost weights chosen by the audit. As shown in Figure 7, across the six alternative weighting schemes, the raw prompt's high-minus-low SES proxy gap ranges from 0.180 to 0.375, while the P2 accessibility-aware prompt ranges from 0.118 to 0.160 and the reranking baseline ranges from 0.111 to 0.153. Thus, the relative ordering of the main DeepSeek mitigation conditions remains stable even when the contribution of transport, equipment, or gym dependence is perturbed.

The sensitivity analysis provides strong evidence for the proxy's internal robustness as a comparative audit measure: the central ordering is not dependent on one specific weight assignment. Small repeated shifts in equipment dependence, transport requirement, and associated cost continue to accumulate into a lower proxy-gap profile under accessibility-aware mitigation. This consistency, together with the score's feature-level traceability, shows that the comparative result is not tied to a single investigator-selected specification and supports reproducible screening. External construct validation is the next step for testing correspondence to human experience.

Together, these analyses provide the empirical basis for answering RQ1–RQ3; the Discussion synthesizes those answers with their theoretical, evidential, and practical implications.

## 7 Discussion

### 7.1 Answers to the research questions

The results provide direct answers to the three research questions. For RQ1, the matched comparisons reveal systematic variation in coded resource assumptions across county-SES contexts: under the raw condition, the high-minus-low access-cost proxy gap is 0.306 even though represented health need is held constant. For RQ2, insurance shifts produce the largest raw scenario-level proxy gap, while county-context shifts retain a residual pattern that captures bundled place-based resource assumptions. For RQ3, accessibility-aware prompting narrows the overall gap to 0.139, and reranking retains that smaller gap while increasing the separate volume-and-intensity alignment rate to 0.250; throughout both mitigation conditions, the structured output schema is preserved and mean personalization remains above 5.1. These findings show how matched isolation, feature-level decomposition, and explicit output selection work together to identify and mitigate socioeconomic variation in resource assumptions.

The six alternative weighting schemes reinforce the comparative ordering, and the common three-model protocol demonstrates the value of model-specific evaluation. The principal remaining uncertainty is external correspondence: expert review, patient-reported feasibility, and implementation studies can determine how the proxy-defined differences map to clinical quality, lived feasibility, adherence, and outcomes beyond the synthetic-profile and ecological-county setting.

Several implications follow from the audit. First, the measured proxy gap concerns cost, transport, equipment, and facility assumptions in the recommendations. The framework offers four complementary audit strengths: its inputs are observable in model outputs, its score is transparent and decomposable, its matched-counterfactual design isolates socioeconomic-context changes while holding represented health need constant, and its main ordering is stable across six alternative weighting schemes. These properties make the framework well-suited to reproducible screening and mitigation comparison. Across SES contexts, the models rarely switch from recommending activity to discouraging it altogether. Instead, they change how activity is operationalized: whether the plan assumes a gym, specialized equipment, reliable transport, or recurring out-of-pocket expense. The audit makes these otherwise diffuse shifts measurable and identifies specific resource assumptions for expert or user-centered follow-up.

Viewed through a socioecological lens, the findings demonstrate that community-level socioeconomic context is reflected in the resource assumptions presented to otherwise matched individuals, making the individual–community interface measurable in model outputs. From a human-centric responsible-AI perspective, the framework supports hybrid oversight: decomposable automated screening directs attention to specific cost, transport, equipment, and facility assumptions, while practitioners and users evaluate clinical appropriateness and lived feasibility. The study thereby operationalizes the individual–community equity link and responsible-innovation principles as a concrete audit-and-mitigation workflow for generative health recommendation (27, 28).

These properties define the proxy's current evidential role: a transparent, reproducible output-audit measure with demonstrated sensitivity to controlled comparisons and robustness to alternative specifications. The present results establish stable differences in the proxy and in the resource assumptions it encodes. Expert assessment, patient-reported feasibility, and implementation studies provide the complementary next layer for testing whether the ordering and magnitude of proxy differences correspond to accessibility experienced by real users. At the present stage, the supported conclusions concern the proposed proxy rather than broader claims about real-world barriers or justice. Transparency and reproducibility establish auditability as an evidential property distinct from construct validity, positioning the proxy for dedicated expert-, user-, and implementation-validation. This complementary architecture provides a well-defined accessibility-oriented component for multidimensional fairness assessment and targeted mitigation. Second, the coarse volume-and-intensity alignment check and accessibility are separate evaluation dimensions. The guideline-constrained condition often produces more standardized and more demanding plans, but it does not reliably remove county-level SES gaps. Conversely, a low-cost recommendation may be clinically inappropriate, while a more costly recommendation may be justified by safety, progression, or individual therapeutic need. The present structured check does not assess frequency distribution, progression, contraindications, or full individualization, so it cannot establish that a recommendation is clinically appropriate or comprehensively evidence based. Accessibility burden should therefore be treated as a feasibility constraint, not as an objective that overrides recommendation quality. Evidence-based exercise prescription requires individualized decisions about the frequency, intensity, time, and type of activity, as well as progression and professional evaluation when indicated (30). A recommendation that requires supervision, equipment, transport, or other resources may be appropriate when those resources are necessary for a safe and effective prescription. Conversely, reducing cost or facility dependence is beneficial only when clinically required dose, progression, contraindication management, and individualization are preserved. Accessibility-aware design should expand the set of feasible evidence-based options rather than replace evidence-based prescription with the least burdensome option. Third, appropriate personalization and a potential proxy-defined accessibility disparity require different counterfactual explanations. A recommendation change that follows an explicit health limitation, stated preference, or safety need can be appropriate even when it increases burden. When represented health need is held constant, a systematic proxy change associated with socioeconomic context identifies a pattern for further evaluation. Human expert and user assessment can then determine whether a specific difference is clinically justified, beneficially personalized, or unfair in practice. The 36-profile cross-model comparison adds a limited model-heterogeneity check. GPT-5.4-mini and Gemini 3.1 Flash Lite Preview exhibit smaller high-minus-low gaps under accessibility-aware prompting, whereas GLM-5 does not. These results motivate model-specific auditing but do not establish general transfer across providers, versions, populations, or deployment settings. The sensitivity analysis strengthens that interpretation. The exact numerical size of the proxy gap depends on how one weights transport, equipment, and gym dependence, which is unsurprising in a proxy-based audit. Yet the qualitative ordering of the main DeepSeek conditions remains stable across all tested alternatives. The mitigation result therefore does not rest on one fragile scoring choice. Instead, it reflects a repeated pattern in how the models operationalize exercise advice under different instructions.

From a public-health perspective, the domain is consequential precisely because fitness advice is often treated as low stakes relative to diagnosis or triage. Yet physical-activity guidance can still encode exclusionary assumptions about neighborhood safety, transport availability, disability accommodation, disposable income, and schedule flexibility. A model that systematically recommends facility-heavy or transport-reliant plans in advantaged contexts may be reflecting ambient social structure rather than responding to genuine differences in need. Fairness evaluation in this domain should therefore inspect explicit cost, transport, equipment, facility, and option-diversity assumptions rather than semantic politeness alone; expert and user evaluation can then determine their lived significance.

Practical feasibility may influence whether a recommendation can be followed, but this audit does not measure adherence. Evidence from physical-activity interventions shows that adherence varies across populations and delivery settings, while behavior-change components such as goal setting, self-monitoring, and feedback may support adherence in some contexts (31, 32). Accordingly, the present results identify access assumptions that may affect implementation; they do not demonstrate that accessibility-aware prompting increases sustained physical activity or improves health. Connecting this audit to physical-activity promotion and exercise-prescription practice will require real-user studies that jointly assess feasibility, prescription quality, behavioral support, and longitudinal outcomes.

Taken together, the matched audit, reranking baseline, cross-model comparison, and score-sensitivity analysis provide reproducible evidence of systematic differences in coded resource assumptions within the tested U.S. synthetic-profile setting. The framework's principal strength is diagnostic: it turns those assumptions into traceable features, reveals where socioeconomic context changes them, and tests whether mitigation changes both mean scores and group gaps. The findings therefore support the proposed proxy as a practical first-stage audit tool. At the present stage, the supported conclusions concern differences on that proxy; complementary expert, community, and real-user evaluation can extend the framework to clinical quality, lived accessibility, justice, and broader fairness.

## 8 Limitations and ethics

This paper is a structured audit of accessibility-related resource assumptions. The user profiles are synthetic, the county context is ecological rather than person-specific, and the scoring framework captures recommendation structure rather than downstream behavior change or clinical outcomes. Synthetic profiles enable controlled counterfactual comparisons, but they do not reproduce the experiences of real users across cultures, languages, disability identities, family structures, healthcare access patterns, or locally specific exercise norms. Several limitations follow directly from this design. County-level context is only a proxy for lived exercise environments, and counties are internally heterogeneous in ways that no ecological measure can fully capture. The SES index is intentionally transparent rather than exhaustive, leaving deprivation, neighborhood safety, and built-environment support only partially represented. The current profile set captures injury limitation and general activity constraints coarsely; injury limitation is not a substitute for disability representation. The audit does not systematically model disability status, assistive-device use, caregiver support, adapted activity needs, or the accessibility of specific exercise environments. The county covariates also do not fully distinguish rural, suburban, and urban settings. The burden weights are heuristic, but they are explicit, decomposable, and open to alternative specifications rather than embedded in an opaque learned evaluator. The six alternative weighting schemes show that the relative ordering of the main DeepSeek conditions is stable under plausible changes to transport, equipment, and gym-dependence weights, supporting the internal robustness of the comparative finding. External validation is the next layer for determining whether score differences correspond to expert judgments, patient-reported feasibility, or observed implementation barriers; accordingly, a one-point difference is not assigned a real-world behavioral meaning. Likewise, the binary volume-and-intensity check omits frequency distribution, progression, individualization, contraindications, and diagnosis-specific prescription requirements. Accessibility, clinical appropriateness, and evidence-based prescription quality therefore remain distinct outcomes. External generalization is limited in two additional ways. The full-scale results come from one model family, and the three-model comparison contains 36 synthetic profiles. It provides a bounded heterogeneity check rather than a representative estimate of behavior across providers or model versions. In addition, all contextual data and guideline thresholds are U.S.-specific. County administration, healthcare access, transport systems, activity infrastructure, cultural norms, and exercise guidance differ across countries and healthcare settings, so the observed burden patterns should not be transferred directly beyond the evaluated U.S. context. Finally, the controlled synthetic-profile design provides clean counterfactual isolation and scalable comparisons, while the next validation layer should connect the transparent output-level proxy to human judgment and behavior. Blinded experts can assess recommendation quality and the relevance of the selected resource features; socio-culturally diverse users can report feasibility across socioeconomic, cultural, and disability contexts; and implementation studies can test whether accessibility-aware prompting improves uptake and sustained adherence without compromising safety or clinical effectiveness. Longitudinal studies can then evaluate health outcomes rather than treating lower burden as a surrogate for benefit. The present evidence establishes reproducible socioeconomic differences in structured recommendation features and a stable comparative response to mitigation within the audited profiles. Ethically, the goal of this work is to identify and mitigate systematic socioeconomic variation in encoded resource requirements, not to endorse autonomous fitness coaching. The manuscript does not recommend using the audited models for medical decision making, and it does not claim that prompt or reranking interventions are sufficient for safe deployment. The narrower implication is still important: systems that generate health-adjacent advice should be audited for socioeconomic variation in explicit resource demands before they are embedded in consumer wellness tools, preventive-health interfaces, or other products likely to reach heterogeneous users at scale.

## 9 Conclusion

The three research questions converge on a coherent conclusion. For RQ1, AI-generated physical-activity advice contains systematic differences in coded cost, transport, equipment, and facility assumptions across matched socioeconomic contexts. For RQ2, insurance and county-context shifts generate the largest proxy differences in the audited profiles. For RQ3, accessibility-aware prompting narrows the county-SES proxy gap, while reranking retains the smaller gap and improves the separate volume-and-intensity alignment check; the structured output schema and high personalization are retained across both mitigation conditions. The bounded cross-model comparison and sensitivity analysis further demonstrate why mitigation should be evaluated model by model and under multiple transparent specifications. The broader contribution is a reproducible first-stage audit framework: matched counterfactuals isolate contextual changes, feature-level scores reveal their source, sensitivity analysis demonstrates stable comparative ordering, and the bounded cross-model panel shows why model-specific evaluation is necessary. Within this intended role, these strengths make the proxy useful for screening and mitigation comparison. The supported conclusion is specific and substantive: the interventions reduce the proposed accessibility-related proxy and its socioeconomic gap in the tested setting. Expert assessment, patient-reported feasibility, and real-world implementation are the next steps for determining whether those proxy improvements translate into genuinely lower accessibility barriers. Lower burden is not inherently a better prescription, and accessibility-aware recommendations should preserve clinically indicated resources and evidence-based exercise principles. Transparency and reproducibility establish auditability as a property distinct from construct validity. This complementary architecture provides an inspectable accessibility-oriented component for comparison, targeted mitigation, and multidimensional fairness evaluation.

### 9.1 Practical implications

The framework offers an actionable pathway for three stakeholder groups. Practitioners and digital-health developers can use the decomposable proxy to flag explicit cost, transport, equipment, and facility demands for review and compare accessibility-aware prompting or reranking before deployment, alongside independent review of clinical quality. Policymakers and responsible-AI governance teams can incorporate socioeconomic-context audits, feature-level reporting, and user validation into evaluation and procurement requirements for health-adjacent AI systems. Researchers can extend the framework with locally calibrated accessibility domains, expert ratings, patient-reported feasibility, disability- and place-sensitive measures, and implementation outcomes. Together, these applications translate the study's diagnostic contribution into a roadmap for equity-oriented, evidence-aligned AI-supported physical-activity guidance.

## Data Availability

The original contributions presented in the study are included in the article/supplementary material, further inquiries can be directed to the corresponding authors.

## Appendix

**Table A1:** Illustrative recommendation shifts across the four health archetypes.

Health archetype	Raw	P2 access-aware	Reduction	% Reduction	Interpretation
Sedentary overweight adult	1.519	1.500	0.019	1.2%	Raw advice is already relatively low-cost, so the P2 accessibility-aware prompt produces only a small access-cost proxy change
Hypertension-risk office worker	1.889	1.685	0.204	10.8%	The P2 accessibility-aware prompt shifts advice toward more home- or walking-based options with fewer facility assumptions
Knee-limited middle-aged adult	3.463	2.611	0.852	24.6%	The largest shift replaces facility-style low-impact resources with more chair-based, home-based, lower-resource alternatives
Older beginner	1.685	1.500	0.185	11.0%	The P2 accessibility-aware prompt reduces coded resource assumptions while preserving beginner-oriented, low-intensity structure

Values are mean access-cost proxy scores under the raw prompt and the P2 accessibility-aware prompt, aggregated across counterfactual scenario types in the DeepSeek audit. Lower values indicate fewer coded cost, transport, equipment, and facility requirements.

### A Prompt template and illustrative case

#### A.1 Prompt structure

The live audit used a structured output request rather than free-form prose. Each prompt contained three components: a system instruction defining the model as a careful public-health-aware fitness coach; a user profile serialized from the synthetic profile table; and an output schema requesting activity fields, weekly dose fields, feasibility fields, and constraint-aware adaptations. Concretely, the schema captured activity types, weekly minutes, intensity, equipment needs, gym dependence, transport requirement, safety cautions, and recommended adaptations. The P2 accessibility-aware variant added an explicit instruction to avoid socioeconomic stereotyping and provide low-cost, low-resource alternatives.

#### A.2 Illustrative recommendation shift

Table A1 extends the illustrative recommendation shift beyond a single profile by summarizing aggregate access-cost proxy changes across the four health archetypes used in the audit. The original knee-limited case remains the largest shift: P2 accessibility-aware prompting lowers its mean access-cost proxy from 3.463 to 2.611, a 24.6% reduction, because the raw outputs more often assume facility-style low-impact resources such as water access or stationary cycling. The other archetypes show smaller but still interpretable changes. Hypertension-risk office-worker and older-beginner profiles move toward lower-cost or more home-based, lower-resource options, whereas the sedentary overweight adult archetype changes little because its raw recommendations already have a comparatively low access-cost proxy. This broader view is consistent with the main finding that P2 accessibility-aware prompting primarily reduces the access-cost proxy without radically changing exercise intent.
